# Development of Bio-Active Patches Based on Pectin for the Treatment of Ulcers and Wounds Using 3D-Bioprinting Technology

**DOI:** 10.3390/pharmaceutics12010056

**Published:** 2020-01-09

**Authors:** Eleftherios G. Andriotis, Georgios K. Eleftheriadis, Christina Karavasili, Dimitrios G. Fatouros

**Affiliations:** Department of Pharmaceutical Technology, School of Pharmacy, Aristotle University of Thessaloniki, GR-54124 Thessaloniki, Greece; gkelefth@pharm.auth.gr (G.K.E.); karavasc@pharm.auth.gr (C.K.); dfatouro@pharm.auth.gr (D.G.F.)

**Keywords:** wound-healing, 3D bio-printing, pectin, propolis, cyclodextrin, 3D bio-inks

## Abstract

Biodegradable 3D-printable inks based on pectin have been developed as a system for direct and indirect wound-dressing applications, suitable for 3D printing technologies. The 3D-printable inks formed free-standing transparent films upon drying, with the latter exhibiting fast disintegration upon contact with aqueous media. The antimicrobial and wound-healing activities of the inks have been successfully enhanced by the addition of particles, comprised of chitosan and cyclodextrin inclusion complexes with propolis extract. Response Surface Methodology (RSM) was applied for the optimization of the inks (extrusion-printing pressure, shrinkage minimization over-drying, increased water uptake and minimization of the disintegration of the dry patches upon contact with aqueous media). Particles comprised of chitosan and cyclodextrin/propolis extract inclusion complexes (CCP), bearing antimicrobial properties, were optimized and integrated with the produced inks. The bioprinted patches were assessed for their cytocompatibility, antimicrobial activity and in vitro wound-healing properties. These studies were complemented with ex vivo skin adhesion measurements, a relative surface hydrophobicity and opacity measurement, mechanical properties, visualization, and spectroscopic techniques. The in vitro wound-healing studies revealed that the 3D-bioprinted patches enhanced the in vitro wound-healing process, while the incorporation of CCP further enhanced wound-healing, as well as the antimicrobial activity of the patches.

## 1. Introduction

There is a vast number of materials available for wound dressing, currently under investigation for different types of wounds and different treatment approaches [1,2,3,4]. The type, depth, and location of a wound, in addition to the extent of damage, the amount of wound exudates and the presence of an infection in the wound site, are key factors in the selection of dressing type. The application of traditional dressings, like cotton bandages or gauzes, absorb the moisture contained by the wound, leading to dehydration of the wound surface, and subsequently decreasing the healing rate. Alternative dressings, fabricated by polymers in the form of films, foams or gels, have already been developed and extensively applied for wound management, as they provide the optimum conditions for wound-healing by maintaining the moisture of the wound and at the same time providing a sense of relief to the patient [3,4].To this end, the aim of this study was the development of a wound-dressing system that could provide an adequate moisture environment under occlusive conditions, and the capability to protect the wound from infection and contamination [5], based on natural, non-toxic materials, like pectin, honey, and propolis.

Pectin belongs to a wider class of materials applied for the fabrication of hydrocolloid dressings, that are well-known to promote wound-healing by maintaining a proper, moist environment 5]. These types of dressing can form a gel upon direct contact with wound exudates, leading to high fluid absorption [6], and at the same time provide an adequate protective barrier against bacterial infection [7]. Pectin serves as a hydrophilic agent that reacts with the wound fluid towards the formation of a soft gel over the wound bed that helps to remove or control exudates. The acidity of the resulting pectin solution enhances the bacterial or viral barrier properties of the system. These wound dressings are well known and extensively studied systems for loading and releasing APIs like antibiotics, analgesics, growth factors and others [7,8].

Pectin has also been studied in combination with honey towards the development of wound dressings [8]. Honey possesses multiple bioactivities related to the wound-healing process and exhibits a broad-spectrum antibacterial activity with variation in potency between different types. Despite the fact that many honey varieties have been studied for their beneficial effects on the wound site, the majority of the research is focused on Manuka honey. The main difference between Manuka honey and other honey varieties is methylglyoxal (MGO), which enhances its antibacterial properties [9]. This honey variety can be diluted by wound exudates (up to 7-fold) and still maintain its inhibition activity against bacteria [10], while its acidity increases the release of oxygen from hemoglobin, creating a less favorable environment for destructive proteases. Additionally, the high osmolarity of honey draws fluid out of the wound bed, creating an outflow of lymph similar to negative pressure wound therapy [10].

Another natural material used in this study is propolis. More than 300 different compounds have been identified in propolis, including aliphatic acids, esters, aromatic acids, fatty acids, carbohydrates, aldehydes, amino acids, ketones, chalcones, dihydrochalcones, terpenoids, vitamins, and inorganic substances, with flavonoids being the most widely studied [11,12]. The positive therapeutic effects of propolis are well established and mostly attributed to the antioxidant activity of polyphenols [11]. It is also well-documented that propolis contains active compounds, like caffeic acid, caffeic phenyl ester, artepillin C, quercetin, resveratrol, galangin, and genistein, that promote cell proliferation [11]. Propolis, in general, is studied for its antiseptic, antibacterial, antimycotic, astringent, spasmolytic, anti-inflammatory, anesthetic, antioxidant, antifungal, antiulcer, anticancer, and immunomodulatory effects. The acceleration of burned tissue repair by propolis is also supported by the literature, and a connection between the ability of flavonoid compounds to reduce lipid peroxidation and prevent necrosis of cells has been suggested [11,13].

Pectin, propolis and Manuka honey could be combined towards the fabrication of wound-healing patches using 3D printing technology. 3D printing is a significant platform that can be used for the production of films and patches in complex geometries, in a controlled manner. 3D printers could be an asset for the development of personalized wound-healing patches to meet the specific needs of individual patients and treatments [14,15]. The fact that 3D bioprinting is a prominent platform for combining cells, growth factors, bio-molecules, and bio-polymers in a controlled manner, makes the use of this technology advantageous compared to traditional techniques for the production of patches, like the solvent-casting method.

In this study, free-standing wound patches that disintegrate in aqueous media were fabricated using a 3D bioprinter. The 3D bioinks were a combination of high methoxylated pectin with Manuka honey, designed either for direct application to the wound site or as free-standing patches after drying. The antimicrobial and wound-healing properties of the fabricated films were enhanced by the addition of propolis inclusion complexes with beta-cyclodextrin. The inclusion complexes were prepared by adding an ethanolic extract of propolis to beta-cyclodextrin. The propolis complexes were further combined with chitosan in order to enhance their adhesion to pectin, by exploiting the ionic bonds formed between the two polymers [16].

## 2. Materials and Methods

### 2.1. Materials

All reagents used were of standard analytical grade. HM pectin from apple (HMP, Degree of esterification = 70–75%, Sigma-Aldrich, Darmstadt, Germany) was used as received. Manuka Honey (MGO 550+, Manuka Health, Auckland, New Zealand) and crude propolis (Propolis from Taygetus Mountain, Green Family-Ellinikon, Thessaloniki, Greece) were kept in the dark at 4 °C and warmed to room temperature prior to use. Beta cyclodextrin (kleptose^®^, Roquette, Zaventem, Belgium) was used as received.

### 2.2. Preparation of 3D Printed Patches

The preparation of 3D-printable pectin inks was realized by the addition of predetermined amounts of apple pectin in 10 mL sterile, double-distilled water, pH 8, to prevent early gelation of pectin. The mixture was left at room temperature for 24 h, under constant magnetic stirring, to ensure wetting and swelling of pectin powder. Subsequently, the temperature of the system was raised to 80 °C under continuous mixing, until a homogeneous viscous mixture was formed (pectin dissolution). Afterward, the pH value of the mixture was lowered (pH 2.6 ± 0.2), by the addition of 20 mg Citric Acid (16.7% *w/v* in double distilled H_2_O), to form a non-freely flowing gel. The gel was cooled to room temperature under continuous mixing, and predetermined amounts of Manuka honey were added. The addition of Manuka honey leads to the formation of harder gels, with a gum-like texture. The prepared 3D-printable pectin inks were loaded to a 3D Bioprinter (CELLINK^®^Inkredible, Gothenburg, Sweden) and films of various geometries were printed, depending on the needs of experiments. Film thickness was determined using a 0–25 mm (±0.01 mm) handheld caliper at five random positions on each film to obtain an average value.

### 2.3. Design of Experiments

Central Composite Design of Experiments (CCDE) was applied with using the Minitab 17.1.0 software (Minitab^®^ 17.1.0, Minitab Inc., State College, PA, USA), for the construction of the design matrixes. In order to quantitatively elucidate the effects of the variables, the responses were subjected to regression analysis, and mathematical models were obtained. Contour plots were constructed based on those models and the preferred experimental conditions were chosen accordingly. The validity of the models is assessed by confirmation experiments contacted in triplicateat the preferred conditions.

### 2.4. Preparation of Propolis Ethanolic Extract (EEP)

The extract was prepared according to a published method [12]. In summary, propolis was cut into small pieces (≈2–3 mm) and mixed with 99% ethanol, at a ratio of 1:10 (propolis:ethanol). The mixture was magnetically stirred for 48 h, in dark at room temperature. The extract was filtered to clarification, and ethanol was removed under reduced pressure. EEP was dissolved in 99% ethanol at a final concentration of 56% *w/v* and stored at 4 °C in an amber vial.

### 2.5. Preparation of Chitosan and β-Cyclodextrin/Propolis Extract Inclusion Complexes (CCP)

Chitosan and β-cyclodextrin/propolis extract inclusion complexes (CCP) were prepared according to literature, with minormodification [13]. Briefly, chitosan (CS) stock solution (1% *w*/*v*) was prepared in aqueous acetic acid solution (2% *v*/*v*) and mixed with an aqueous solution of β-CD (5% *w*/*v*), at predetermined ratios. EEP solution was added dropwise, and the final mixture was sonicated using a probe sonicator (SONICS, vibracell™, Newtown, CT, USA), in an ice bath, to prevent thermal decomposition of EEP. Ethanol was removed under reduced pressure and CCP particles were collected after freeze-drying. Freeze-dried CCP was grounded using a mortar and a pestle, and passed through a 125 μm mesh. The fine powder was gradually added to the 3D-printable pectin ink, under continuous mixing, until a homogeneous ink was formed.

### 2.6. Determination of Total Polyphenol Content

The total polyphenol content of CCP particles was determined according to the Folin–Chiocaltau method [17]. Briefly, CCP samples (10 mg) were suspended in 200 μL ethanol and placed in a rotary mixer for 1 h, in the dark and at room temperature. After the extraction of propolis from CCP, the mixture was centrifuged at 4500 rpm, and 50μL of the supernatant was added to a solution containing 50 μL of theFolin–Chiocaltau reagent, 700 μL H_2_O and 200 μL of 10% *w/v* sodium carbonate solution. The solution was placed for 1 h at room temperature and color development was measured at 650 nm. The color development intensities of the sample extracts were expressed as Gallic Acid Equivalent per mg of sample (mg GAE/mg).

### 2.7. Dynamic Light Scattering and ζ-Potential Measurements

Dynamic light scattering (DLS) and ζ-potential measurements were performed using a Malvern Nanosizer ZS, Malvern Instruments (UK). The droplet size, polydispersity index (PDI), and z-potential values were recorded in triplicate and evaluated.

### 2.8. Film Swelling Studies

Rectangular patches (1 × 1 cm) were printed and left to dry at 75 °C until at a constant weight. The patches were weighted and placed in 50 mL PBS for 60 s, under mild agitation. Subsequently, the swollen patches were withdrawn, gently blotted with filter paper and weighted. The swelling properties were expressed as the percentage weight gain over the initial dry weight. The patches were then left to dry at 75 °C until the weight variations were stabilized, and reweighed to calculate the mass loss during the swelling test.

### 2.9. 3D Printing Shape Fidelity Assessment

**Figure 1:**
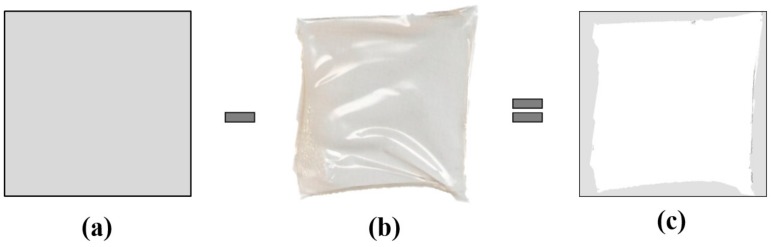
Theoretical dimentions of the patches (**a**), experimental dimensions of the 3D-printed patches after drying (**b**), and their difference (**c**), as it is calculated by Equation (1).

The shape fidelity of the 3D-printed patches was quantified based on the variance between the theoretical and experimental dimensions of the 3D printed patches, after drying. Briefly, 3D-printed patches (2 × 2 cm) were fabricated and left to dry until a constant weight was achieved at 75 °C. The dimensions of the dry patches were compared to a computer-designed control frame (2 × 2 cm), using a conventional desktop 2D scanner (HP DesckJet 2630, Hewlett-Packard, Palo Alto, CA, USA) (Figure 1) and an image analysis software (ImageJ public domain software, NIH, Bethesda, MD, USA). The deviation from the expected shape is expressed as Equation (1),

F(%) = 100 × (Ac − As)/Ac(1)

where Ac and As are the projection areas (in pixels) of the control frame and the 3D-printed patch, respectively.

### 2.10. Determination of Film Opacity

Film opacity was determined according to previously reported studies. Rectangular strips of 3D-printed Pectin films (3d Pec), containing various amounts of CCP, were printed according to the geometry of a UV–Vis cuvette. The 3D printed patches were then placed in contact with the inner wall of the cuvette, and the absorption spectrum of the patches was obtained from 400–700 nm in a UV-Visible spectrophotometer (Shimadzu 1601, Kyoto, Japan). Film opacity was defined as the area under the curve, divided by the film thickness and expressed as Absorbance Units × nanometers/millimeters (AU × nm/mm). All measurements were conducted in triplicate.

### 2.11. Optical Microscopy

All 3D-printed patches were observed under an optical microscope (Celestron MicroDirect 1080p HD Handheld Digital Microscope, Celestron, Torrance, CA, USA) at a magnification of 220×.

### 2.12. Determination of Relative Surface Hydrophobicity

The relative surface hydrophobicity of the films was estimated by the sessile drop method, based on the optical contact angle method [18,19]. A 2 μL droplet of ethylene–glycol was deposited onto the patch surface. Contact angle measurements were carried out using a digital microscope (CelestronMicroDirect 1080p HD Handheld Digital Microscope). The digital camera of the microscope was placed horizontally to capture the droplet image. Image analysis software (ImageJ public v1.52a, NIH, Bethesda, MD, USA) was used to measure the angle between the patch surface and the tangent to the drop of liquid, at the point of contact with the film surface. Seven parallel measurements were performed for each film at 25 °C.

### 2.13. Mechanical Tests

The tensile properties of the formulated films were investigated using a TA-XT2i instrument (Stable Micro Systems, Godalming, Surrey, UK) (*n* = 3). To achieve this, dumbbell-shaped films with 50 × 10 × 0.2 mm dimensions were 3D-printed. Thickness was measured using a caliper, and an average value of five points was obtained. The fabricated films were fixed onto the clamps of the instrument, and tensile tests were performed under a speed of 0.5 mm/s, to determine the tensile strength and the elongation at break [20].

### 2.14. Biodhesion Studies

The bioadhesion performance of the films was examined using a TA-XT2i instrument (Stable Micro Systems, Godalming, Surrey, UK) (*n* = 3). Freshly excised porcine skin was supplied by a local slaughterhouse. Porcine skin was used as anex vivomodel for bioadhesion studies [21]. The skin was attached onto PET films with cyano-acrylate glue, to avoid the extensive deposition of glue residues on the instrumentation, and subsequently fixed onto the platform of the device with double adhesive tape. The films were mounted onto the probe using double-adhesive tape. The performance of the films was assessed under dry conditions, to simulate the direct use of the films on a dry wound. Alternatively, 100 μL of PBS or liquid 3D pectin ink were instilled onto the skin, to simulate the application of the films onto a wet wound surface or the indirect utilization of the films as a support dressing on wounds, covered with the 3D pectin ink. A force of 1 N was applied, to maintain contact of the formulations with the skin for 60 s, and the probe was withdrawn at a speed of 1 mm/s [22]. The maximum force (F_max_) of detachment was recorded, whereas the work of adhesion (W_ad_) was determined from the area under the curve of the force-versus-distance plot.

### 2.15. Determination of Antimicrobial Properties

A medium pouring method was used to determine the antimicrobial activity of the 3D-printed patches containing CCP against two bacterial strains (*Staphylococcus aureus* and *Escherichia coli*) [23]. Three-dimensional-printed patches (discs with diameter 1 cm, height 0.15 mm and weight 40 mg) were sterilized by UV light (30 W, 1.0 m) for 30 min. The sterile patches were added to 15 mL polypropylene tubes, containing 1 mL 10^7^ CFU/mL bacterial suspension, diluted in 9 mL sterile saline solution. After the complete dissolution of the patches, the solution was cultivated in sterile plates. All the plates were incubated at 37 °C for 24 h, and the colony number was measured. Bacteriostasis was calculated by Equation (2),

Q(%) = 100 × (A − B)/A(2)

where Q, A, and B represent the growth inhibition, the mean colony count (CFU/mL) of the control group and the mean colony count (CFU/mL) of the sample group, respectively.

### 2.16. Film Disintegration Test

An ERWEKA Disintegration Tester (ERWEKA GmbH, Langen, Germany) was used to measure the disintegration time of the 3D-printed films, modifying the USP30<701> Disintegration procedure, using 900 mL of distilled water at 37 °C. Disks of 1 cm diameter and 0.12 mm average thickness were printed and placed into individual tubes of the basket-rack assembly. Time was recorded until complete erosion of the films.

### 2.17. Scanning Electron Microscopy

The morphological features of the 3D-printed patches were assessed using a Zeiss SUPRA 35VP SEM microscope (Zeiss GmbH, Oberkochen, Germany). Samples were placed on aluminum stubs and coated with 15 nm gold, using an Emitech K550X DC sputter coater (Emitech Ltd. Ashford, Kent, UK) apparatus, prior to imaging.

### 2.18. Fourier-Transform Infra-Red (FTIR).

The chemical structure of the 3D printed patches was confirmed by recording the FTIR spectra (IR Prestige-21, Shimadzu, Japan). The resolution of the spectrum was set at 4 cm^−1^. The recorded wavenumber range was 800–4000 cm^−1^, and 64 spectra were averaged. The commercially available software IR Solutions (Shimadzu, Japan) was used to process the spectral data.

### 2.19. Cell Culture

Dermal human fibroblast cells (HDFa, ATCC^®^ PCS-201-012™) were cultured in 96-well plates containing high-glucose DMEM with 10% *v/v* heat-inactivated fetal bovine serum (FBS) and 1% (*v*/*v*) antibiotic antimycotic at 37 °C in a humidified atmosphere of 5% CO_2_ [24].

### 2.20. Cell Viability—MTT Assay

The viability of HDFa cells was evaluated using the MTT assay based on the ability of viable cells to convert thiazolyl blue tetrazolium bromide solution to purple formazan crystals in their mitochondria [25,26]. The HDFa cells were seeded into 96-well plates and incubated with three different concentrations of dissolved patches containing different concentrations of CCP (0.1, 1 and 5 mg/mL 3DPect of 0, 2.5, 5, 10, 20 and 30% CCP *w*/*w* of dry film, respectively) for 24 and 48 h. The culture medium was then removed, and the cells were rinsed three times with PBS. The MTT agent (5 mg/mL in PBS) was added into the wells and the plates were incubated at 37 °C for 3 h. Sequentially, formazan crystals were dissolved in DMSO and the absorbance of each well was measured at 540 nm. Cell viability was calculated using Equation (3),

Cell Viability (%) = 100 × (A_S_ − A_B_)/(A_C_ − A_B_) (3)

where A_S_, A_C_, and A_B_ are the absorbance values of the sample well, control well and blank well, respectively.

### 2.21. Cell Scratch Assay (In Vitro Wound Healing)

The cell scratch assay was proposed for the assessment of the wound-healing properties of the 3D-printed patches, as a facile and low-cost in vitro method to evaluate the migration ability of cells [24]. Briefly, HDFa cells were seeded into 6-well plates, at an initial density of 2 × 10^5^ cells/well and incubated at 37 °C for 72 h (in high glucose DMEM with 10% *v/v* FBS). Two perpendicular scratches were generated in each well, via a sterile tip of a pipette (200 μL), as well as an intersection at a point close to the center of the well. The cross-shaped scratch was used as a location marker. After rinsing with PBS, the cells were incubated with three different concentrations of dissolved 3D-printed patches (0.1, 1 and 5 mg/mL 3DPect) and serum-free medium. The distribution and quantity of cells in the scratch area in each well were monitored under a microscope, and images were recorded at 0, 24 and 72 h, respectively. Wound width and wound closure were calculated according to Equations (4) and (5),

Relative Wound Width (%) = 100 × W_f_/W_i_ (4)

where, W_i_ and W_f_ are the initial and final wound width, respectively.

Relative Wound Closure (%) = 100 × (A_i_ − A_f_)/A_i_ (5)

where, A_i_ and A_f_ are the initial and final cell-free area of the simulated wound, respectively.

Wound width was calculated as the average distance between the edges of the scratch. Manual quantification was carried out and the initial and final wound width was calculated as the average of 50 width measurements across the scratch. The wound area was calculated based on the cell-free area in captured images using the ImageJ public domain software (NIH, Bethesda, MD, USA).

### 2.22. Statistical Analysis

All experiments are reported in triplicate, and statistical analysis was performed for data derived from different samples. All results are expressed as mean ± standard deviation and checked by normality tests. OriginLab v9.0.0software (Originlab Corporation, Wellesley Hills, MA, USA) was used for statistical analysis. Student’s paired *t*-test was performed to compare the data of paired samples. A value of *P* < 0.05 indicated statistical significance.

## 3. Results and Discussion

### 3.1. Process Optimization

#### 3.1.1. Optimization of 3D Printable Pectin Inks

CCDE was performed, based on Manuka honey/pectin ratio and pectin concentration. The design matrix and the corresponding responses are listed in Table 1. Six centerpoints have been selected (runs 1, 3, 7, 10, 13, and 14) and the corresponding responses are listed in Table 1. The differences between the values of the response for the centerpoints are indicative of the system variability, caused mainly by the addition of undiluted Manuka honey. The direct use of viscous materials like honey induces experimental variations, mainly due to the intense gelation of pectin at honey-rich regions of the mixture. These variations are surpassed by the continuous mixing of the system until homogeneity is reached. These alterations have an impact on the measured responses and they are taken under consideration by the Response Surface Methodology process. The following equations (Equations (6)–(9)) were obtained by regression analysis (using the full quadratic model) of the swelling properties of the patches (S), the mass loss of the patches after swelling (D), the shape fidelity of the patches (F) and the maximum pressure needed for the ink to be extruded through the printer nozzle (P), relating X_1_ and X_2_ (Manuka honey/pectin ratio and pectin concentration, respectively):

P (kPa) = 27.5 − 697 × (X_2_) + 302 × (X_1_) + 5781 × (X_2_)^2^ − 5162 × (X_1_)^2^ + 3400 × (X_2_) × (X_1_), (*R*^2^ = 0.894) (6)

F (%) = 30.98 − 119 × (X_2_) − 46 × (X_1_) + 429 × (X_2_)^2^ + 1817 × (X_1_)^2^ − 1357 × (X_2_) × (X_1_), (*R*^2^ = 0.679) (7)

S (%) = 662 − 5000 × (X_2_) − 6289 × (X_1_) + 14,032 × (X_2_)^2^ + 15,158 × (X_1_)^2^ + 34,733 × (X_2_) × (X_1_), (*R*^2^ = 0.421) (8)

D (%) = 82.2 − 1119 × (X_2_) + 149 × (X_1_) + 3968 × (X_2_)^2^ − 1679 × (X_1_)^2^ + 174 × (X_2_) × (X_1_), (*R*^2^ = 0.709) (9)

**Figure 2:**
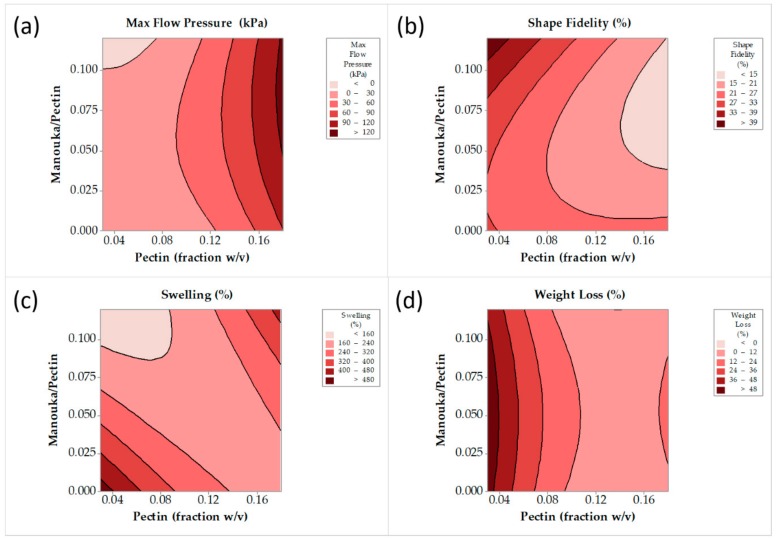
Maximum flow pressure (**a**), shape fidelity (**b**), film swelling (**c**) and film weight loss (**d**) contour lines against pectin concentration and Manuka honey/pectin ratio.

Contour plots have been constructed in order to visualize the aforementioned models (Equations (6)–(9)). Figure 2a shows a distinct area of moderate working extrusion pressure (20–120 kPa), that is considered ideal for the printing process. Figure 2b shows a distinct minimum shape-deviation from the theoretical dimensions of patches, for a pectin concentration 0.125–0.18 *w/v* of ink, and Manuka honey/pectin ratio over 0.05 *w*/*w*. Figure 2c shows two maxima of film-swelling for a pectin concentration less than 0.075 *w/v* of ink and more than 0.15 *w/v* of ink and for a Manuka honey/pectin ratio less than 0.025 *w*/*w* and more than 0.1 *w*/*w*. Both of these maxima are located to extreme areas that are not preferred due to intense ink gelation (high Manuka honey concentration) or insufficient ink gelation (low Manuka honey concentration). Finally, Figure 2d shows a minimum film weight loss for a pectin concentration over 0.1 *w/v* of ink, depending almost exclusively on pectin concentration. Based on the visualization of the trends of the responses, as they are depicted by the above contour plots, the selected value for pectin concentration was 0.13 *w/v* of ink, and the value for Manuka honey/pectin ratio was 0.08 *w*/*w*. The model describing the swelling properties of the films failed to fit the data properly, and it was not taken into consideration for the final selection.

#### 3.1.2. Optimization of Chitosan and Cyclodextrin/Propolis Extract Inclusion Complexes

Table 2 summarizes the design matrix and the corresponding responses. The responses were subjected to regression analysis (using full quadratic model) and the following equations (Equations (10)–(13)) were obtained for particle size (d_nm_), zeta potential (ζ), PDI and EEP-loading, based on Gallic acid equivalent, as determined by the Folin–Chiocaltau method (GAE), relating X_1_ and X_2_(EEP and chitosan concentration, respectively):

d_nm_(nm) = 880 − 1 × (X1) − 115 × (X2) − 14.6 × (X1)^2^ + 601 × (X2)^2^ − 8 × (X1) × (X2), (*R*^2^ = 0.608) (10)

ζ (mV) = −40.6 + 27.3 × (X1) + 201.2 × (X2) − 1.89 × (X1)^2^ − 100.5 × (X2)^2^ − 23.41 × (X1) × (X2), (*R*^2^ = 0.859) (11)

PDI = 0.350 + 0.0407 × (X1) − 0.097 × (X2) + 0.0006 × (X1)^2^ + 0.200 × (X2)^2^ − 0.0837 × (X1) × (X2), (*R*^2^ = 0.367) (12)

GAE (μg/mg) = −13.6 + 18.0 × (X1) + 42.7 × (X2) − 3.37 × (X1)^2^ − 45.3 × (X2)^2^ − 0.5 × (X1) × (X2), (*R*^2^ = 0.147) (13)

**Figure 3:**
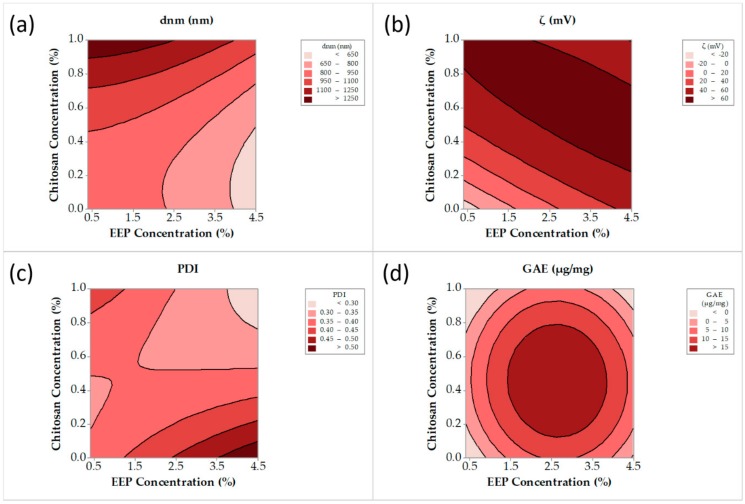
Particle size (**a**), zeta potential (**b**), polydispersity index (PDI) (**c**) and Gallic acid equivalent (**d**), contour lines versus propolis ethanoic extract (EEP) and chitosan concentration.

Contour plots have been constructed to visualize the aforementioned models (Equations (10)–(13)). Figure 3a shows a distinct minimum of particle size for an EEP concentration more than 3.8% *v/v* and a chitosan concentration less than 0.45% *w*/*v*. A broad range of chitosan and EEP concentrations exhibited zeta potential values greater than 40 mV (Figure 3b). The PDI (Figure 3c) was less than 0.4 (narrow distribution) for a broad range of chitosan and EEP concentrations. Finally, Figure 3d shows a distinct maximum of GAE for EEP concentration in the range of 1.5–4.5% *v/v* and chitosan concentration in the range 0.2–0.8% *w*/*v*. Based on the visualization of the trends in the responses, as they are depicted by the above contour plots, the selected value for EEP concentration was 4.5% *v/v* and the valuefor chitosan concentration was 0.6% *w*/*v*. The models describing the PDI and GAE for the EEP concentration of the CCP failed to fit the data properly and they were not taken into consideration for the final selection.

#### 3.1.3. Confirmation Experiments

Response Surface Methodology was chosen as a statistical tool to screen experimental conditions through the visualization of the trend of the responses. The obtained models from the regression analysis were used for the construction of the contour plots and subsequently for the selection of the formulation, according to the depicted trends. Models that do not fit the experimental data (low *R*^2^ values), were not taken into account for the selection of the final experimental conditions. The selection of the experimental conditions was performed by setting the desirable limits of the final responses based on the contour plots. In order to confirm the validity of the selected experimental conditions, additional experiments were carried out. The maximum flow pressure, shape fidelity, film swelling, and film weight loss for the printed patches, along with the particle size, *ζ*-potential, PDI and Gallic acid equivalent for the CCP particles, were obtained for the selected conditions, as listed in Table 3 and Table 4, respectively. The desirable limits (Target Limits), along with the theoretical and experimental values of the responses, are listed in Table 3 and Table 4. The reproducibility of the results for the selected conditions within the desirable limits validates that the RSMwas a useful approach for screening and selecting the experimental conditions.

### 3.2. Evaluation of 3DPectin Patches Containing CCP

The optimum ink formulation was mixed with the optimally prepared CCP, under various final CCP concentrations (0%, 2.5%, 5%, 10%, 20% and 30% *w*/*w* CCP based on the amount of dry pectin). The prepared inks were loaded to the bioprinter, and the resulting patches were dried at room temperature to achieve a constant weight of the formulations. The free-standing films were stored in a desiccator, at room temperature.

#### 3.2.1. Film Opacity

**Figure 4:**
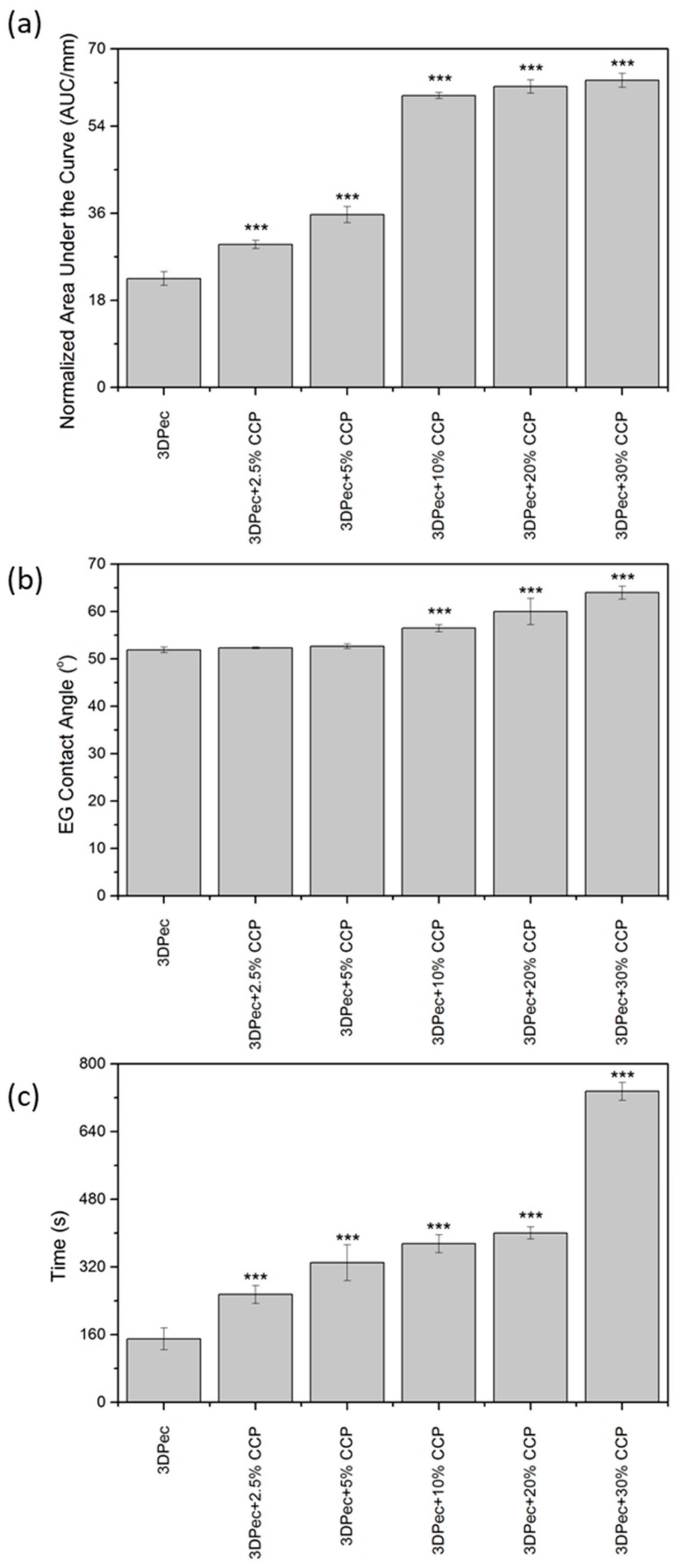
Data of (**a**) film opacity, (**b**) relative surface hydrophobicity, and (**c**) film disintegration time, obtained for patches with different concentrations of CCP. *** *P* < 0.05 vs. control.

**Figure 5:**
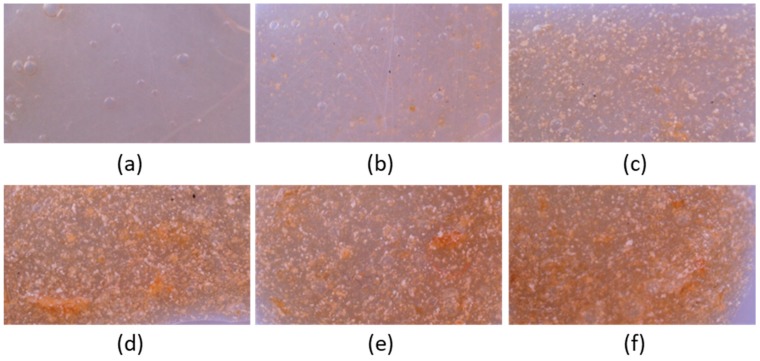
Optical microscopy images of 3D-printed pectin films with:(**a**) 0%, (**b**) 2.5%, 5%, (**c**) 10%, (**d**) 20% and (**e**,**f**) 30% *w*/*w* CCP.

The 3D pectin patches with different amounts of CCP were assessed for their opacity. Transparent wound dressings permit the inspection of a wound without the need for dressing removal, and thus they are recommended for application on superficial and shallow wounds with low exudates [4]. The opacity quantitative analysis of the patches is shown in Figure 4a, while the actual patches are shown in Figure 5 (optical microscopy with 220× magnification). Patches, comprised of 0%, 2.5% and 5% *w*/*w* of CCP, are considered transparent (Figure 5a–c) with an increasing opacity correlated to higher concentrations of CCP. CCP-free patches were visually transparent, while the presence of CCP in the films resulted in a darker and more yellow appearance. As shown in Figure 4a and Figure 5, the film opacity of the 3D-printed pectin films was relatively low, exhibiting an increase in relation to CCP incorporation. The notably higher opacity of the CCP-containing films, compared to the control, is attributed to their extensive light scattering, due to the inhomogeneous film structure, originating from CCP, which aggregates, expanding over the continuous pectin-rich phase. A key factor that influences the extent of dispersion of CCP in the film matrix, and consequently the opacity of the films, is the interaction between pectin and chitosan (present in CCP). The different charges of pectin and chitosan lead to the formation of ionic bonds that directly result in the topical cross-linking of pectin around CCP [14], thus inhibiting extended dispersion of the later. This topical cross-linking can elucidate the presence of the larger coagulants that were visible in the films.

#### 3.2.2. Relative Surface Hydrophobicity

Low contact angles, less than 90°, measured for CCP-free and composite films, are typical of hydrophilic materials, like pectin films. CCP incorporation up to 5% *w*/*w* into the pectin films does not influence the hydrophilic characteristics of the resulting film surface (*P* > 0.05), as was deduced from the data for contact angles presented in Figure 4b. On the other hand, CCP incorporation higher than 5% *w*/*w* into the pectin films affected the hydrophilic character of the film surface (*P* < 0.05). Despite the statistical significance of this effect, the film surface was still considered hydrophilic, with contact angles less than 65°. These negligible alterations to the hydrophobicity of the patches suggest that hydrophobicity is preferentially influenced by the changes in surface roughness following CCP incorporation, rather than by chemical or compositional changes to the surface. In conclusion, the increase in the hydrophobicity of the film surface is considered artifactual, as the ethylene–glycol droplet used for the contact angle measurements is considered to exclusively contact the pectin matrix.

#### 3.2.3. Film Disintegration Test

A disintegration test of the 3D-printed patches is shown in Figure 4c. The incorporation of CCP into the films is suggested to influence the disintegration time of the patches (*P* < 0.05), compared to the control film. The disintegration time presented an increasing trend, proportional to the addition of CCP, with a six-fold increase in the case of films comprising 30% *w*/*w* CCP, compared to the control group. This increase is solely attributed to cross-linked pectin moieties, due to the formation of ionic bonds with the incorporated chitosan molecules.

#### 3.2.4. Film Surface Observation

**Figure 6:**
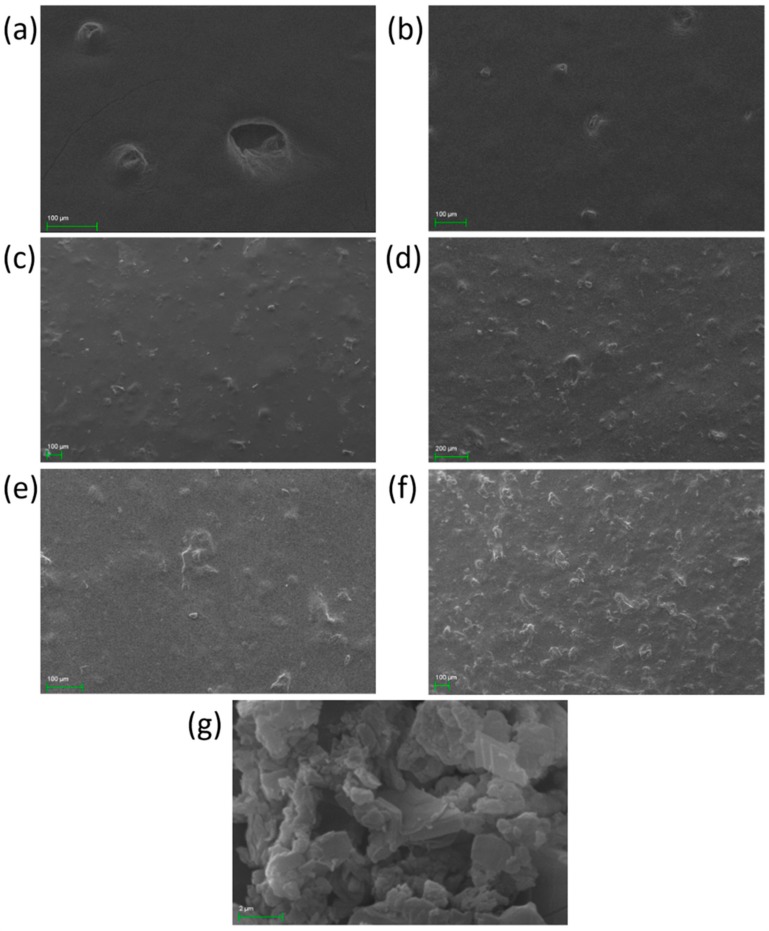
SEM images of 3D-printed pectin films with: (**a**) 0%, (**b**) 2.5%, 5%, (**c**) 10%, (**d**) 20% and (**e**,**f**) 30% *w*/*w* propolis extract inclusion complexes (CCP); and (**g**) CCP powder.

The films’ surface was observed by optical microscopy and SEM (Figure 5 and Figure 6). Both techniques revealed the presence of printing-related defects on the surface of the control film. These defects are characterized by the presence of voids, formed during the printing process, which gradually decreased in size and resulted in a bubble-like formation. The inhomogeneity of the composite films, as is depicted in Figure 5b–f, is attributed to the presence of CCP coagulants, with increasing intensity as the concentration of CCP is increased. The transparency and the color of the films are clearly depicted in Figure 5, where films with more than 5% *w*/*w* CCP appear to be opaque with a distinct, dark yellow color, due to propolis. Finally, the CCP micrograph shows the structure of propolis-containing particles that are smaller than those incorporated in the films, indicating the presence of intense coagulation due to topical cross-linking.

#### 3.2.5. Fourier-Transform Infra-Red (FTIR) 

**Figure 7:**
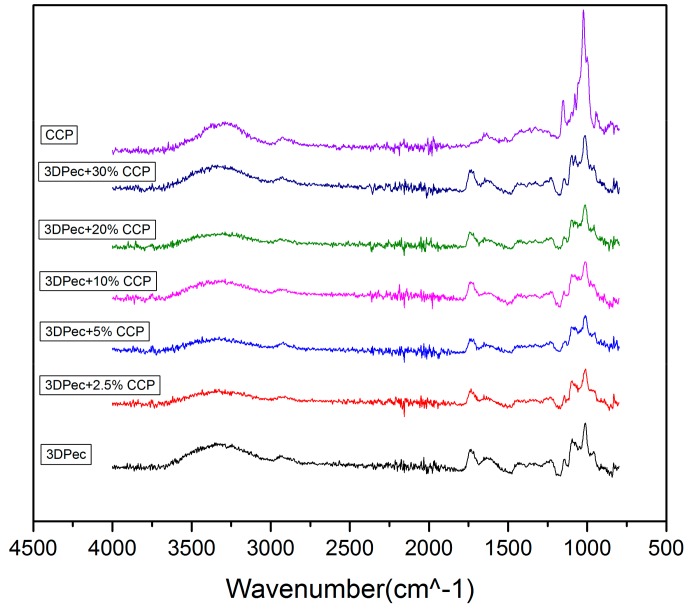
ATR-FTIR Spectra of 3D-printed pectin films with: 0%, 2.5%, 5%, 10%, 20% and 30% *w*/*w* CCP; and CCP powder.

The chemical structure of the surface of the 3D-printed patches was confirmed by recording their ATR-FTIR spectra. Figure 4, summarizes the ATR-FTIR spectra of the patches, the spectra of their composites with CCP, and the spectra of CCP. The spectra in Figure 7 revealed a broad band around 3700–3000 cm^−1^ for all films and for CCP, typical for polysaccharides, attributed to the O–H stretching vibration (OH), while peaks around 3000–2800 cm^−1^ were attributed to C–H stretching vibrations (νCH) [14]. Regarding the spectrum of pectin films, two bands were detected between 1800–1500 cm^−1^, which were attributed to the stretching vibrations of the carbonyl group (νC=O) [14]. The band located at 1733 cm^−1^ corresponds to the νC=O of the methyl ester group (COOCH_3_) and to the non-dissociated carboxyl group (COOH), while the band located at 1624 cm^−1^ is assigned to the asymmetric stretching vibration of the carbonyl group of the carboxyl ion (COO–) [14]. The CCP spectrum revealed a band centered at 1647 cm^−1^, attributed to the νC=O of the amide group (also known as Amide I band) of the acetylated units of chitosan, present in the CCP. The almost identical spectra of the control 3D-printed pectin films and the composite films with CCP are indicative of the surface composition of the films that are mainly composed of a pectin matrix, while CCP particles are coated with pectin to such an extent that the presence of free CCP on the surface is considered negligible.

#### 3.2.6. Mechanical Tests

The tensile properties of the prepared films are presented in Table 5. It has been reported that the mechanical properties of wound dressings are key to the optimal applicability of the formulations on the skin and the avoidance of mechanical abrasion [27,28]. The incorporation of propolis-based microparticles resulted in significant alterations in the tensile strength of the specimens (*P* < 0.05). The maximum tensile performance was recorded in the case of the formulation with 2.5% *w*/*w* CCP, presenting values of tensile strength and elongation of approximately 50 N/mm^2^ and 2.7% *w*/*w*, respectively. Formulations with a higher content of CCP reduced tensile properties, due to the simultaneous incorporation of chitosan, as it has been documented that chitosan-based films exhibit poor mechanical properties [29]. Furthermore, it was hypothesized that large amounts of the particles in the ink induced the formation of low-homogeneity structures, as was previously discussed, thus affecting the mechanical properties of the films. The results for the Young Modulus of the fabricated pectin films show an increase, following the incorporation of CCP, from 7.76 to 9.1 N × mm^−2^ (*P* < 0.05). Films with 2.5%, 5%, and 10% *w*/*w* CCP have similar Young Modulus values, in contrast with the films with 20% and 30% *w*/*w* CCP, which are stiffer. The loss of elasticity, due to CCP incorporation, is also attributed to the cross-linking that takes place between pectin and chitosan moieties of the CCP.

#### 3.2.7. Bioadhesion Studies

The bioadhesive performances of the formulated films, under dry and wet conditions, are reported in Table 6. Negligible adhesion was observed under dry conditions, in all cases. The instillation of PBS onto the porcine skin induced a significantly higher adhesion performance (*P* < 0.05). Further studies were conducted with the incorporation of liquid pectin ink onto the porcine skin. The experiments revealed greater F_max_ and W_ad_ values for all specimens, compared to PBS.

Regarding the content of microparticles in the films, alterations in the bioadhesion parameters were further recorded, due to the simultaneous incorporation of the bioadhesive chitosan [30,31]. Up to a certain amount of CCP, a significant enhancement in the bioadhesive performance of the specimens was evidenced (*P* < 0.05), as the formulation comprising 20% *w*/*w* CCP exhibited F_max_ and W_ad_ values of approximately 6.4 and 8.8 N × mm, respectively. In line with the tensile tests, however, the higher content of CCP reversely affected the bioadhesive performance of formulation, with 30% *w*/*w* CCP, suggesting the limited homogeneity of the films.

#### 3.2.8. Determination of Antimicrobial Properties

**Figure 8:**
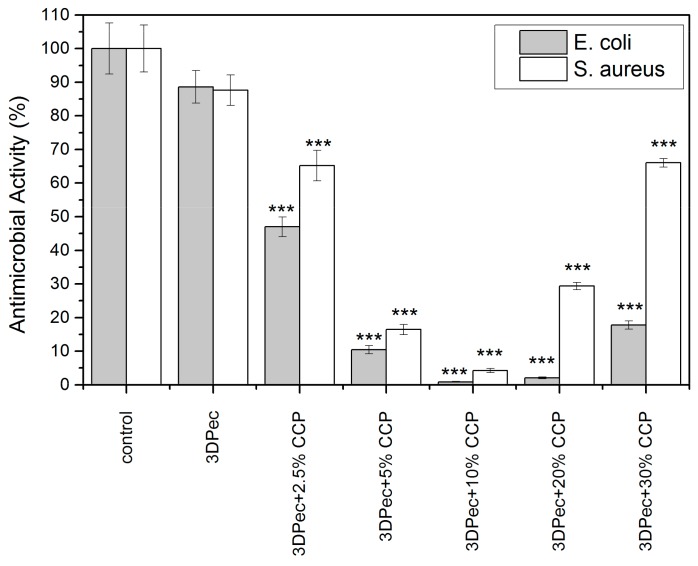
Antimicrobial activity of 3D-printed pectin films with: 0%, 2.5%, 5%, 10%, 20% and 30% *w*/*w* CCP, against two bacterial strains of interest. *** *P* < 0.05 vs. control.

The antimicrobial properties of the 3D-printed films, comprised of pectin and Manuka honey, and their composites with CCP, were evaluated using the medium-pouring method. Figure 8 shows the percentage of microbial viability in the presence of dissolved patches. The results show that 3D-printed pectin films exhibit insufficient antimicrobial activity, despite the presence of manuka honey. This was an expected outcome, as the amount of honey in contact with the bacteria is highly diluted (more than 10-fold). On the other hand, CCP incorporation of up to 10% *w*/*w* CCP is followed by an intense increase of more than 95% in the antimicrobial activity of the films. Notably, the antimicrobial activity decreased for 20% and 30% *w*/*w* of CCP, presenting a minimum for CCP concentrations of 10% *w*/*w*. It was evidenced that the presence of insoluble CCP clusters, which were formed by the topical cross-linking of pectin with chitosan, affect the extent of the contact area between the propolis-containing particles and the bacteria incorporated into the aqueous media.

#### 3.2.9. Cell Viability Assay

**Figure 9:**
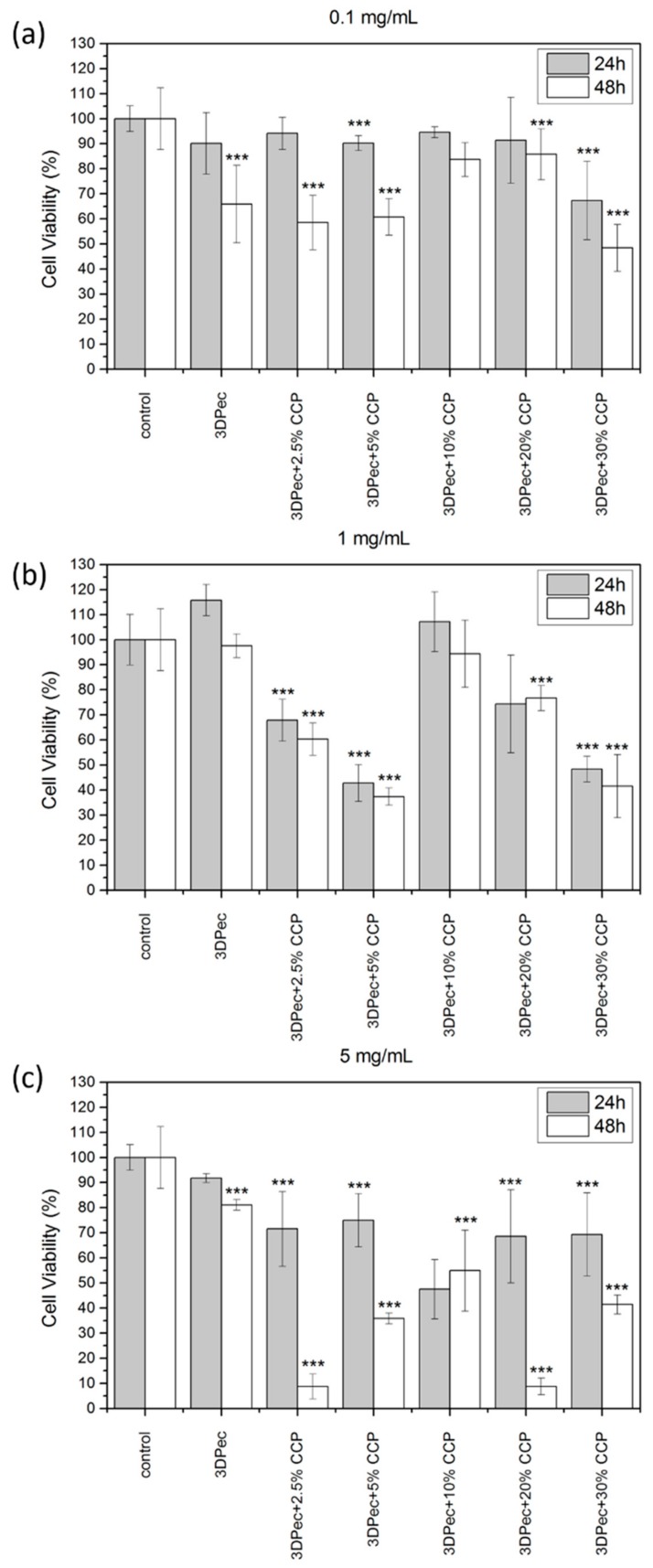
Cell viability assay of the 3D-printed pectin films with: 0%, 2.5%, 5%, 10%, 20% and 30% *w*/*w* CCP, for (**a**) 0.1 mg/mL, (**b**) 1 mg/mL and (**c**) 5 mg/mL of dissolved patches in the cell culture medium. *** *P* < 0.05 vs. control.

The viability results of HDFa cells, treated with 3D-printed patches that contain different amounts of CCP, as well as for three different concentrations of the dissolved patches in the cell culture medium, are summarized in Figure 9. For the low concentration of dissolved patches in the cell culture medium (0.1 mg/mL), cell viability was not significantly affected for the first 24 h; however, decreased viability was evidenced at 48 h, estimated at 50% of the control culture. Additionally, cell viability was slightly reduced with increasing the CCP concentration in the films. This behavior was attributed to the addition of pectin films that resulted in a possible decrease in the pH of the medium, combined with an increase in the medium viscosity that is believed to affect cell proliferation. Another key factor that affects cell viability is the presence of insoluble material (especially for films containing higher amounts of CCP) that are capable of physically obstructing the cells, by either covering or immobilizing them. This phenomenon was more intense for higher concentrations of dissolved patches in the cell culture medium (1 and 5 mg/mL), where the decrease in cell viability was higher for the first 24 h, followed by the lack of a distinct pattern correlating CCP concentration with cell viability. The absence of a distinct pattern of cell viability for concentrations of 1 and 5 mg/mL of dissolved films in the cell culture medium is attributed to the inhomogeneous character of the films containing CCP, due to the presence of the topical cross-linking of pectin with chitosan. This immediately resulted in the presence of more insoluble material in the cell culture medium, and subsequently a more intense inhibition of cell growth.

#### 3.2.10. Cell Scratch Assay (In Vitro Wound Healing)

**Figure 10:**
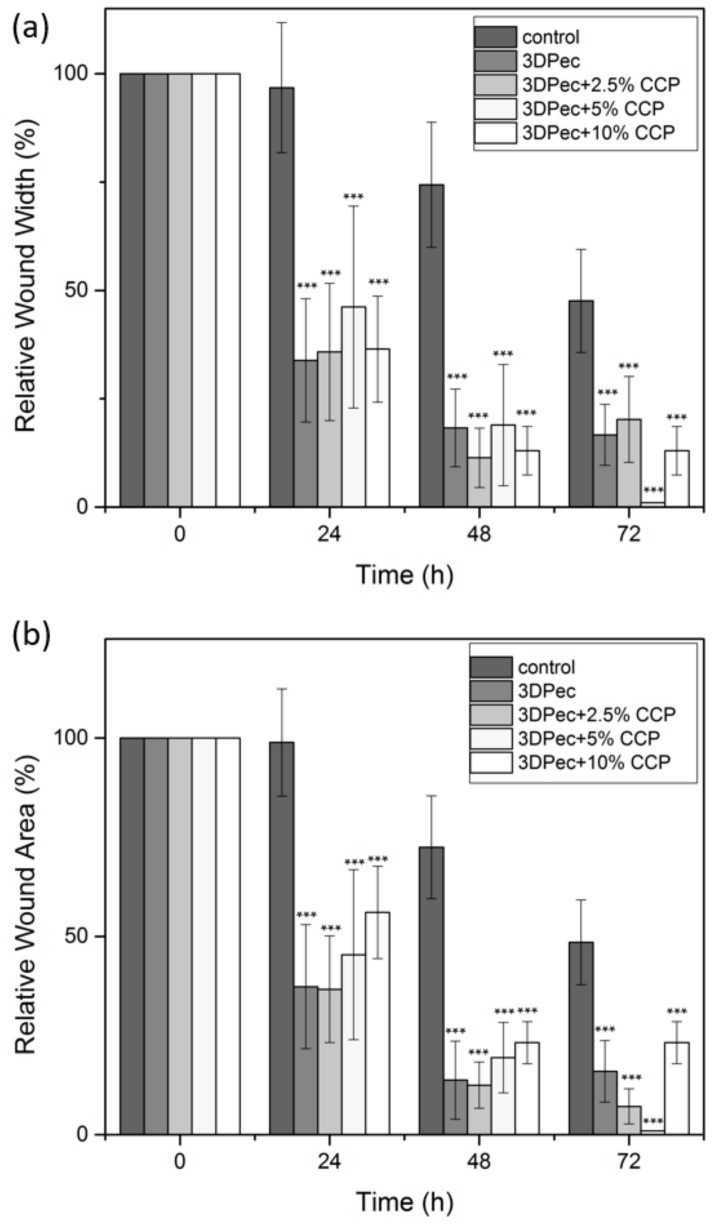
(**a**) Relative Wound Width and (**b**) Relative Wound Area calculated by the in vitro wound-healing assay for 3D-printed pectin films with: 0%, 2.5%, 5%, 10%, 20% and 30% *w*/*w* CCP, for 0.1 mg/mL of dissolved patches in the cell culture medium. *** *P* < 0.05 vs. control.

**Figure 11:**
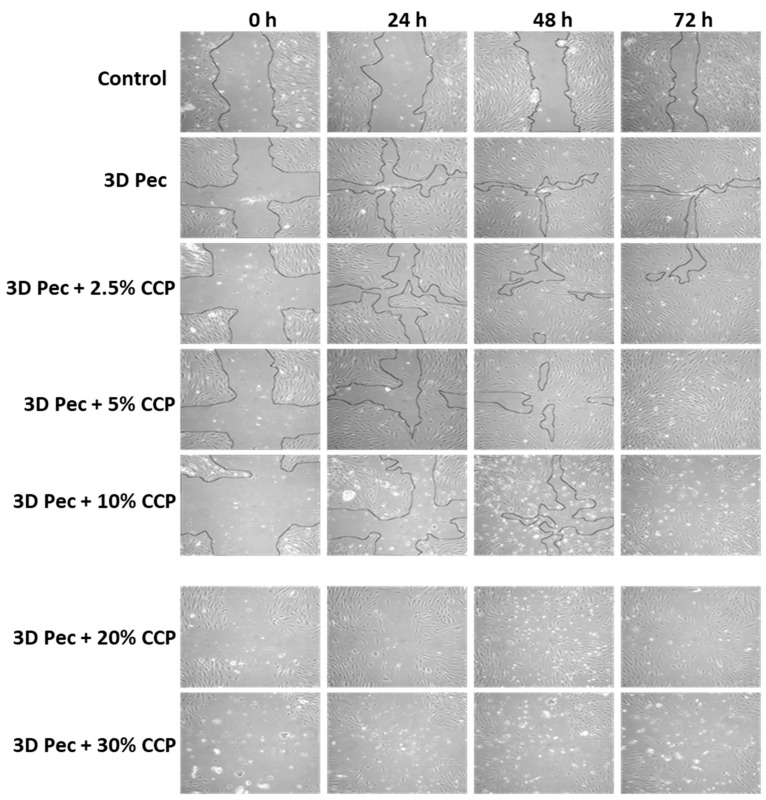
Time-lapse of in vitro wound healing process for 0.1 mg/mL of the dissolved patches in the cell culture medium (100× magnification).

The cell scratch assay was applied for assessment of the wound-healing potential of the 3D-printed patches. The scratch assay was applied to different amounts of CCP and for three different concentrations of the dissolved patches in the cell culture medium (0.1, 1 and 5 mg/mL). HDFa cells were cultured, and a cross-shaped scratch was generated on the cell monolayer, simulating a wound. The wound closure in the presence of 3D-printed pectin patches containing CCP was monitored for 72 h. Figure 10a,b shows the relative wound width and the relative wound area, respectively. Figure 11 shows the time-lapse of the actual wound healing process. The results of the in vitro wound-healing assay revealed that the 3D-printed pectin patches exhibit strong wound-healing properties in vitro. This behavior is further enhanced by the addition of CCP (up to 5% *w*/*w*), where full closure (Relative Wound Width = 0% and Relative Wound Area = 0%) of the simulated wound was evidenced. In all cases, elongated cell formations with multiple infusions were observed, typical of migrating cells, along with changes in their spatial conformation. For CCP concentrations higher than 10% *w*/*w*, the presence of an insoluble film material exhibited an inhibitory effect on cell migration, despite the migration of individual cells towards the empty scratched area. The higher concentrations of dissolved patches in the cell culture medium presented a similar behavior to the samples containing 0.1 mg/mL of patches (with 20% and 30% *w*/*w* CCP), and thus they are not presented. It is suggested that the discrimination efficacy of the assay was compromised by higher amounts of insoluble film debridement, and thus the deduction of a safe conclusion was arbitrary.

## 4. Conclusions

Biodegradable, pectin-based 3D-printable inks have been developed as an efficient treatment approach for direct and indirect wound-dressing applications. The evaluation methodology followed in the current study rendered the inks suitable for 3D-printing applications. Free-standing transparent films that disintegrate upon contact with aqueous media were developed via 3D-bioprinting. The antimicrobial and wound-healing activities of the fabricated dressings were investigated and effectually enhanced by the incorporation of particles comprised of chitosan and cyclodextrin inclusion complexes with propolis extract.

## Figures and Tables

**Table 1:** Central-composite design matrix defining conditions for printing patches by using 3D pectin inks, together with the experimental values of the respective responses.

Run	Variables in Uncoded Unit		P (kPa)	F (%)	S (%)	D (%)
	Manuka honey/pectin (*w*/*v*)	Pectin (*w*/*v*)				
1	0.05	0.10	32	5.8	230	4.0
2	0.00	0.15	40	21.2	180	8.8
3	0.05	0.10	33	19.1	200	11.3
4	0.00	0.05	10	28.3	600	31.8
5	0.10	0.15	73	16.4	180	4.4
6	0.10	0.05	9	37.1	250	25.6
7	0.05	0.10	32	15.4	240	9.1
8	0.12	0.10	20	25.1	170	11.4
9	0.05	0.03	10	23.9	120	65.1
10	0.05	0.10	30	28.8	240	13.0
11	0.00	0.10	25	23.3	200	7.6
12	0.05	0.18	134	14.5	340	10.4
13	0.05	0.10	20	16.9	190	4.4
14	0.05	0.10	60	17.5	180	6.5

**Table 2:** Central-composite design matrix defining conditions for the production of CCP, together with the experimental values of the respective responses.

Run	Variables in Uncoded Unit		d (nm)	ζ (mV)	PDI	GAE (μg/mg)
	EEP (%)	Chitosan (%)				
1	1	0	760	−26.8	0.35	2.57
2	2.5	0.5	1080	61.1	0.29	3.82
3	0.4	0.5	1140	59.8	0.41	0.23
4	4.5	0.5	720	60	0.42	5.07
5	2.5	0.5	780	59.5	0.42	5.00
6	4	1	1080	55.4	0.26	2.67
7	2.5	0	1130	44.1	0.62	4.41
8	4	0	510	32.7	0.44	7.77
9	2.5	0.5	850	55.6	0.37	3.35
10	1	1	1250	54.6	0.38	0.52
11	2.5	1	1360	55.2	0.41	1.51
12	2.5	0.5	650	59.6	0.25	5.09
13	2.5	0.5	750	61.2	0.31	8.8
14	1	0	760	−26.8	0.35	2.57

**Table 3:** Confirmation experiments executed for the selected conditions for 3D pectin inks.

EEP (%)	Chitosan (%)	Parameter	d (nm)	ζ (mV)	PDI	GAE (μg/mg)
4.5	0.6	**Target Limit**	650–800	>60	0.30–0.35	5-10
		**Theoretical Value**	715	65	0.33	7.9
		**Experimental Value**	715 ± 8	62 ± 1.8	0.4 ± 0.11	5.1 ± 0.2

**Table 4:** Confirmation experiments executed for the selected conditions for CCP particles.

Manuka Honey/Pectin	Pectin	Parameter	P (kPa)	F (%)	S (%)	D (%)
0.08	0.13	**Target Limit**	30–60	15–21	160–240	0–12
		**Theoretical Value**	62	17	204	7
		**Experimental Value**	35 ± 4	14 ± 5	220 ± 16	12 ± 8

**Table 5:** Data obtained from the mechanical tests for the tensile strength, elongation at break, and Young Modulus of the fabricated 3D-printed pectin films with different amounts of CCP.

Formulation	Tensile Strength (N × mm^−2^)	Elongation (%)	Young Modulus (N × mm^−2^)
**3DPec**	29.68 ± 3.05	1.91 ± 0.08	7.76 ± 0.47
**3DPec + 2.5% CCP**	49.62 ± 1.71	2.72 ± 0.22	9.14 ± 0.43
**3DPec + 5% CCP**	43.65 ± 2.23	2.35 ± 0.03	9.28 ± 0.36
**3DPec + 10% CCP**	36.96 ± 2.79	1.91 ± 0.06	9.67 ± 0.43
**3DPec + 20% CCP**	35.62 ± 1.62	1.63 ± 0.13	10.95 ± 0.38
**3DPec + 30% CCP**	36.31 ± 3.22	1.59 ± 0.11	11.41 ± 0.22

**Table 6:** Data obtained from the bioadhesion studies of the fabricated 3D printed pectin films with different amounts of CCP, for direct contact with the tissue (dry), after wetting the tissue with double distilled H_2_O (wet) and after wetting the tissue with 3D-printable pectin ink (ink).

Formulation	Conditions	F_max_ (N)	Wad × 10 (N × mm)
**3DPec**	Dry	0.05 ± 0.03	0.07 ± 0.04
	Wet	0.48 ± 0.10	0.68 ± 0.13
	Ink	1.29 ± 0.11	3.44 ± 0.21
**3DPec + 2.5% CCP**	Dry	0.06 ± 0.02	0.13 ± 0.03
	Wet	0.85 ± 0.15	1.48 ± 0.34
	Ink	1.39 ± 0.08	5.38 ± 0.42
**3DPec + 5% CCP**	Dry	0.14 ± 0.07	0.52 ± 0.11
	Wet	1.59 ± 0.11	5.67 ± 0.37
	Ink		
**3DPec + 10% CCP**	Dry	0.17 ± 0.04	0.64 ± 0.14
	Wet	1.04 ± 0.09	2.28 ± 0.24
	Ink	1.47 ± 0.09	5.27 ± 0.32
**3DPec + 20% CCP**	Dry	0.26 ± 0.03	0.99 ± 0.16
	Wet	2.88 ± 0.14	6.43 ± 0.28
	Ink	3.66 ± 0.15	8.79 ± 0.35
**3DPec + 30% CCP**	Dry	0.17 ± 0.12	0.32 ± 0.19
	Wet	1.38 ± 0.25	2.76 ± 0.54
	Ink	2.04 ± 0.33	6.16 ± 0.98
